# Gut mucosal microbiota and local intervertebral disc immunity: candidate circulating-cytokine-independent pathways informed by Mendelian randomization evidence

**DOI:** 10.3389/fimmu.2026.1938023

**Published:** 2026-09-22

**Authors:** Rui Chen, Deduo Xu, Yuhua Li, Jun Yang

**Affiliations:** 1 Department of Orthopedics, Ruijin Hospital, Shanghai Jiao Tong University School of Medicine, Shanghai, China; 2 Department of Orthopedics, The First Naval Hospital of Southern Theatre Command, Zhanjiang, Guangdong, China; 3 Department of Pharmacy, Second Affiliated Hospital of Naval Medical University, Shanghai, China; 4 Department of Pharmacy, The First Naval Hospital of Southern Theatre Command, Zhanjiang, Guangdong, China

**Keywords:** bacterial extracellular vesicles, gut mucosal immunity, gut-disc axis, immune privilege, intervertebral disc degeneration, local immune microenvironment, Mendelian randomization, microbial metabolites

## Abstract

Chronic low back pain secondary to intervertebral disc degeneration (IVDD) is the leading cause of disability across all musculoskeletal disorders worldwide. For decades, gut-disc crosstalk research has been framed almost exclusively by a systemic inflammatory paradigm, in which circulating pro-inflammatory cytokines (e.g., TNF-α, IL-1β) act as obligate mediators linking gut microbial dysbiosis to intervertebral disc degeneration. However, multi-cohort two-sample Mendelian randomization (MR) analyses have not identified significant mediation by the circulating inflammatory factors tested to date, exposing a gap in current mechanistic frameworks. A null indirect effect indicates that the measured mediators did not statistically explain the exposure-outcome association; it does not exclude a contribution from untested systemic pathways. Two core protective commensal taxa, *Akkermansia muciniphila* (Akk) and *Eubacterium coprostanoligenes*, have been genetically validated to reduce IVDD risk, yet their beneficial effects cannot be explained by systemic immune activation. Focusing on mucosal immunology and tissue-localized immune homeostasis, this review integrates human genetic data, *in vitro* nucleus pulposus experiments, *in vivo* bacterial extracellular vesicle (EV) evidence, and clinical endplate microbiome profiles to delineate three candidate gut mucosa-derived immune regulatory axes that are not explained by the circulating cytokines measured to date: (1) microbe-derived soluble metabolites—which are themselves circulating mediators—reshape local disc metabolic and immune responses via epigenetic modulation and receptor signaling; (2) EVs secreted by *A. muciniphila* act as cross-barrier immune messengers targeting the avascular disc tissue; (3) gut bacterial fragments translocate across damaged endplate barriers to trigger site-specific immune remodeling without elevating systemic inflammatory markers. This three-pathway framework offers one possible reconciliation of the null mediation findings from MR studies with the collapse of intrinsic disc immune privilege. Each proposed axis remains incompletely validated, and the available evidence does not establish that they operate independently of untested systemic immune pathways. We further summarize methodological limitations of gut-spine microbiome research and outline mucosa-targeted microbial immunotherapies for chronic low back pain. Collectively, this framework helps shift the field away from over-reliance on systemic inflammation as the sole mechanistic explanation for IVDD pathogenesis.

## 1 Introduction: mucosal microbiota, barrier function, and local immune regulation in intervertebral disc degeneration

Globally, low back pain (LBP) imposes an annual economic burden of USD 90–134.5 billion and remains the top musculoskeletal cause of disability in the general population. With a lifetime prevalence as high as 80%, LBP progresses to chronic pain in approximately 5–10% of affected individuals, posing a substantial medical and socioeconomic burden (1). Driven by population aging, the incidence of spinal degenerative disorders continues to rise annually. In 2019, the global prevalence of osteoarthritis exceeded 500 million, and the number of people living with IVDD surpassed the same threshold; unfortunately, current interventions only alleviate symptoms and fail to reverse the degenerative process (2, 3).

Modic changes (MCs)—characteristic signal alterations of the lumbar endplate and bone marrow visible on magnetic resonance imaging—represent an independent risk factor for severe disabling low back pain, and their pathological core lies in endplate barrier disruption and dysregulation of the local immune microenvironment (4). Colonization of the intervertebral disc by low-virulence microbes is closely associated with MCs and severe IVDD; notably, these low-virulence organisms only trigger mild local immune activation without elevating systemic inflammatory markers (5). Degenerative lumbar diseases share a common degenerative cascade and immune remodeling process, and inflammation-mediated pain mechanisms have long been a core research target in the field (6). Two-sample Mendelian randomization, a method that mitigates confounding bias inherent to cross-sectional studies, has been applied to identify causal links between specific gut microbiota taxa and spinal stenosis as well as IVDD (7).

The long-dominant hypothesis of the gut-disc axis rests entirely on the single systemic inflammatory chain of “intestinal leakage–LPS-TLR4-NF-κB”: gut microbial dysbiosis disrupts tight junctions in the intestinal mucosal epithelium, allowing lipopolysaccharide (LPS) to enter the circulation and activate the TLR4 signaling pathway, which in turn stimulates peripheral immune cells to secrete pro-inflammatory factors including TNF-α, IL-6 and IL-17. These factors are then delivered to the intervertebral disc via the bloodstream to induce nucleus pulposus matrix degradation. However, existing observational studies can only confirm a correlation between microbial dysbiosis, IVDD and chronic low back pain; they cannot rule out confounding variables or reverse causation, making it difficult to establish a robust causal evidence chain (8). The concept of the “gut-disc axis” has been formally proposed in multiple reviews. The healthy intervertebral disc is an avascular, immune-privileged tissue, and after barrier breakdown during degeneration, gut microbial signals can regulate disc homeostasis at a distance. Yet most prior reviews have heavily emphasized the systemic inflammatory cascade, with comparatively limited discussion of tissue-localized immune regulatory mechanisms within the disc microenvironment (9).

Evidence for tissue-localized microbial signals is, however, beginning to emerge. Metabolomic analysis of human lumbar discs has identified bacterial-specific metabolites within the nucleus pulposus, including gut flora-associated oxylipins and quorum-sensing molecules, consistent with microbial colonization and local microenvironment remodeling in degenerated discs (10). A network pharmacology and molecular docking study identified six core microbial metabolites—butyrate, propionate, acetate, succinate, trimethylamine oxide and 3-indolepropionic acid—with IL6, TLR4, CXCL8 and JUN as core targets; 3-indolepropionic acid showed the strongest binding affinity (11). These are in silico predictions that require biological validation. Intestinal Clostridium sporogenes metabolizes dietary tryptophan to indole propionic acid, which suppresses osteoclast differentiation and protects against bone loss in ovariectomized mice via pregnane X receptor signaling (12). Whether homologous indole-mediated regulation occurs in the intervertebral disc has not been tested.

In contrast to the traditional emphasis on systemic inflammatory cytokines, recent large-scale Mendelian randomization evidence points to blood metabolites as quantifiable mediators in the gut microbiota–IVDD axis. Liu et al. (2025) integrated the MiBioGen consortium, GWAS Catalog of blood metabolites, and FinnGen R10 data to dissect causal pathways and identified that specific metabolites significantly mediated the effects of *Lachnoclostridium, Ruminococcaceae* UCG003, and *Eubacterium fissicatena* group on intervertebral disc degeneration, with mediated proportions ranging from 8.1% to 18.1% (13). Nevertheless, the overall picture remains incomplete. Other large-scale MR studies have likewise identified causal associations between gut microbial taxa and IVDD or related disc disorders, but did not formally test mediation by circulating inflammatory factors (14–16). Such null mediation results are consistent with, but do not positively demonstrate, the involvement of local or mucosal pathways; limited instrument strength, statistical power, mediator coverage, pleiotropy, phenotype definition and multiple testing all warrant consideration. Thus, while metabolite-level mediation is now genetically supported, existing reviews still suffer from homogeneous mechanisms and one-sided evidence, over-relying on a single systemic inflammatory explanatory pathway and neglecting mucosal immunity and local tissue-specific regulation within the disc microenvironment. There is an urgent need to construct a new mechanistic framework that integrates systemic metabolic signals with local immune–microenvironment interactions, rather than attributing IVDD solely to circulating inflammation.

Moving beyond the traditional single systemic inflammatory immune framework, this review takes three independent classes of microbe-derived cross-organ immune regulatory pathways as its core line of argument: ① soluble microbial metabolites (SCFAs/tryptophan derivatives/secondary bile acids) mediate local immune homeostasis regulation; ② *Akkermansia*-derived bacterial extracellular vesicles (EVs) act as cross-barrier immune messengers; ③ *in situ* immune remodeling driven by gut bacterial fragments translocating across damaged endplates. We use “candidate circulating-cytokine-independent” to denote pathways not statistically explained by the circulating cytokines measured to date, not pathways that are non-inflammatory: TLR activation, NF-κB signaling, macrophage polarization and Th17/Treg imbalance are themselves inflammatory processes (17–19). MR analyses have identified two protective gut microbial taxa: the *Eubacterium coprostanoligenes* group (OR = 0.81, P = 3.0×10^−5^, significant even after Bonferroni correction) (20) and *Akkermansia muciniphila* (OR = 0.973, 95% CI 0.950–0.996, P = 0.02) (18). These two estimates derive from different exposure and outcome datasets (**Table 1**). Increased abundance of both taxa significantly reduces the genetic risk of IVDD, and they serve as the core targets for mechanistic validation in this review. The full text follows a three-tier progressive logic: “mucosal microbiota synthesizes immune signals → vesicle cross-barrier transport → *in situ* immune response in the intervertebral disc”.

**Table 1:** Suggestive causal associations between gut microbiota and intervertebral disc degeneration (results from Mendelian randomization IVW method).

Bacterial taxon	Effect direction	OR (95% CI)	P value	Reference
*Eubacterium coprostanoligenes* group (genus)	Protective	0.81 (0.73-0.89)	(3.0×10^-5^)	(20)
Rhodospirillaceae (family)	Protective	0.92 (0.87-0.98)	0.008	(20)
Rhodospirillales (order)	Protective	0.93 (0.87-0.98)	0.014	(20)
Bifidobacterium (genus)	Protective	0.92 (0.85-0.99)	0.029	(20)
Ruminococcaceae UCG011 (genus)	Protective	0.94 (0.89-0.99)	0.030	(20)
Prevotella9 (genus)	Protective	0.94 (0.88-1.00)	0.048	(20)
Sutterella (genus)	Risk	1.18 (1.08-1.29)	0.0004	(20)
Marvinbryantia (genus)	Risk	1.17 (1.07-1.29)	0.001	(20)
Escherichia-Shigella (genus)	Risk	1.11 (1.01-1.23)	0.030	(20)
Verrucomicrobia (phylum)	Risk	1.09 (1.01-1.18)	0.035	(20)
Allisonella (genus)	Risk	1.06 (1.00-1.11)	0.037	(20)
*Akkermansia* (genus)	Protective	0.973 (0.950-0.996)	0.020	(18)

P<0.05 indicates a suggestive association. Only the Eubacterium coprostanoligenes group approaches the Bonferroni correction threshold (P<2.37×10^−4^). These results aggregate findings from two independent published MR analyses and were not generated from a single unified analytical pipeline. Data for the 11 taxa listed above are sourced from reference (20); the *Akkermansia* row is sourced from reference (18). Exposure GWAS: MiBioGen consortium (n=18,340; 211 taxa) (29) for the 11 taxa, and a population-based cohort of 5,959 individuals with genotypic and metagenomic records for *Akkermansia*. Outcome dataset: FinnGen (29,508 cases and 227,388 controls) for the 11 taxa, and FinnGen (41,669 cases and 294,770 controls) for *Akkermansia*.

The intact intervertebral disc has traditionally been regarded as an immune-privileged, near-sterile tissue. This view has been challenged: 16S rRNA sequencing of MRI-normal discs from brain-dead organ donors detected a rich bacterial community, with 58 bacterial taxa shared between the gut and disc microbiomes and 6 common to gut, skin and disc (21). Whether such signals represent true commensals, contamination, or translocation remains unresolved, and endplate barrier damage may nevertheless increase bacterial ingress. *Cutibacterium acnes* detected in surgical specimens must be strictly differentiated between skin contamination strains and gut translocated strains, as the two differ significantly in biofilm formation capacity and immunogenicity (21). It should be noted that 16S rRNA sequencing from low-biomass disc specimens is susceptible to kit-derived contamination; therefore, these data cannot definitively prove gut-origin microbial translocation. As early as 2001, a study first detected *Propionibacterium acnes* in herniated disc tissue, launching research on disc microbes and local immunity and providing the earliest clinical evidence for the gut bacterial translocation hypothesis (22). Drawing on population genetic evidence from Mendelian randomization, *in vitro* nucleus pulposus cell experiments, extracellular vesicle research and Modic endplate clinical microbiome data, this review constructs a three-tier regulatory model of mucosal microbiota–cross-barrier transport–*in situ* disc immunity. The three candidate pathways—none of which has been shown to operate independently of systemic immunity—are summarized in **Figure 1**.

**Figure 1:**
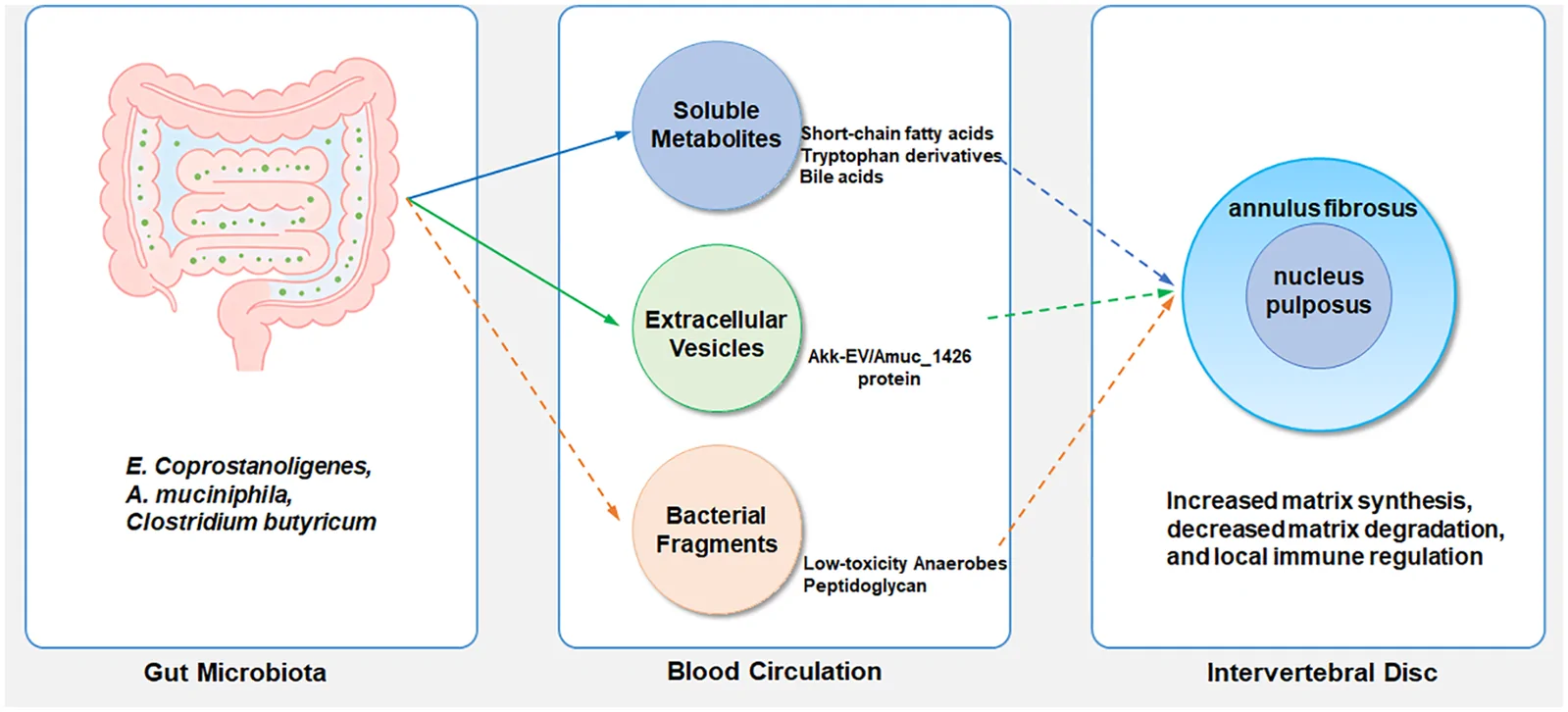
Multi-layer cross-organ immune signaling network derived from intestinal mucosal symbionts: candidate circulating-cytokine-independent pathways. Left panel lists two core protective gut taxa identified by multi-cohort Mendelian randomization—*Akkermansia muciniphila* and *Eubacterium coprostanoligenes*—alongside butyrate-producing *Clostridium* strains. Three candidate signal carriers traverse the circulation (middle panel): small-molecule soluble metabolites, bacterial extracellular vesicles, and low-virulence peptidoglycan-containing microbial fragments. They are proposed to act on the avascular, immune-privileged intervertebral disc (right panel). Solid arrows: steps supported by direct experimental or genetic evidence; dashed arrows: inferential, unproven steps. Full textual discussion of unresolved inferential steps is provided in the main text.

Building on this global pathway model, the following sections re-examine the single systemic inflammatory hypothesis that has dominated the field for years, supported by multi-cohort Mendelian randomization genetic data.

## 2 Limitations of the systemic inflammatory immune paradigm: dissociation between circulating and local immune responses

To systematically challenge the dominant systemic inflammation paradigm, this section first summarizes the core evidence chain of the traditional hypothesis, then integrates multi-cohort mediation MR data, clinical cohort results and animal experimental evidence to examine whether local disc immune damage is dissociated from circulating inflammatory factors. All causal arguments are grounded in a standardized two-step mediation Mendelian randomization statistical framework. To clearly illustrate the full logic of the genetic analysis and the multi-layer sensitivity validation design, the study workflow is summarized in **Figure 2**. Based on the complete stratified mediation analysis design in **Figure 2**, multiple independent large-sample genetic cohorts have yielded consistent negative mediation conclusions, which are demonstrated below from the perspectives of MR genetic evidence, clinical cohorts and animal experiments. The traditional systemic inflammatory immune pathway was the core explanatory basis for early gut-disc axis research, and the hypothesized evidence chain can be summarized as follows: gut microbial dysbiosis induces increased permeability of the intestinal mucosal epithelial barrier (intestinal leakage), circulating LPS enters the bloodstream and activates the TLR4 signaling pathway, stimulating the body to secrete large amounts of circulating pro-inflammatory immune factors such as TNF-α and IL-1β, which ultimately induce extensive degradation of the nucleus pulposus extracellular matrix (23). *In vitro*, human nucleus pulposus cells stimulated with 10 ng/mL LPS for 24 h show TLR4 upregulation and NF-κB activation, with increased secretion of TNF-α, IL-1β and IL-6 and reduced aggrecan and type II collagen, providing cellular-level support for this hypothesis (24). A classic review in the field systematically sorted out the role of cytokines in intervertebral disc degeneration, further consolidating the theoretical framework of systemic inflammatory immune-driven degeneration (25). However, this single *in vitro* stimulation model cannot replicate the bidirectional immunomodulatory effects of microbiota *in vivo*, nor can it explain the negative mediation results found in subsequent genetic studies.

**Figure 2:**
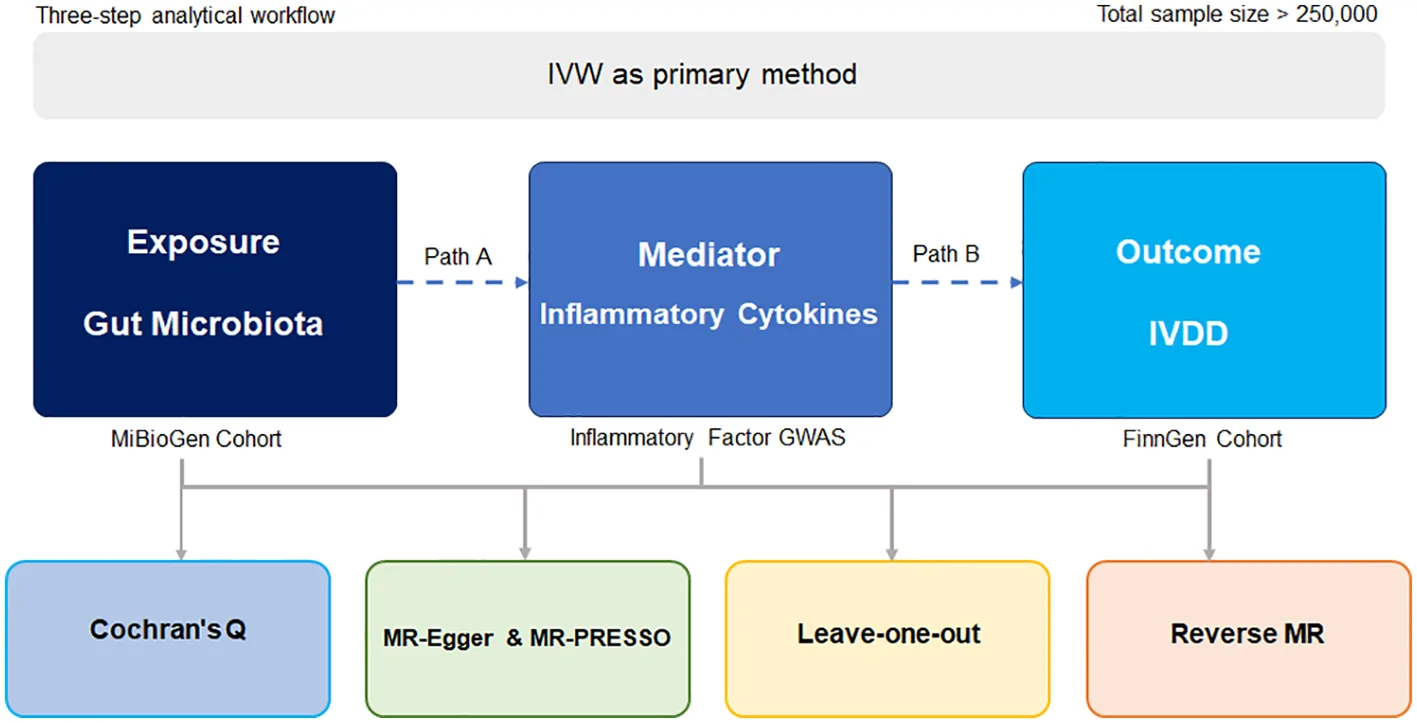
Study design of two-step mediation Mendelian randomization. This figure presents a standardized MR framework for mediation-effect testing, showing exposure (Gut Microbiota), circulating inflammatory mediator (Inflammatory Cytokines), and outcome (IVDD) layers with corresponding GWAS datasets labelled. Dashed arrows denote causal research hypotheses under statistical testing (Path A, Path B and total causal effect); solid arrows represent established sensitivity-analysis workflows. Four sensitivity-analysis modules are displayed below, with an aggregate sample size > 250 000.

Multiple two-sample Mendelian randomization studies have not found a statistically significant mediating role for the systemic circulating immune factors tested at the population genetic level. This supports insufficient evidence for mediation by the measured circulating factors, rather than positive evidence for dissociation between local and systemic immune responses. By contrast, a 2026 two-step mediation Mendelian randomization study of 473 gut microbial taxa, 91 circulating inflammatory proteins and three degenerative lumbar spine disorders (intervertebral disc disorders, degenerative spondylolisthesis and lumbar spinal stenosis; FinnGen R12) identified 13 genetically supported putative microbiota–inflammatory protein–degenerative lumbar spine disorder pathways, with mediation proportions ranging from 7.55% to 13.22% across key pathways; IL-6 and IL-18 were convergent mediators for intervertebral disc disorders, with IL-6 and IL-20RA showing risk-increasing and IL-18 a protective association that remained robust after multiple-testing correction (26). The same study additionally performed *in vivo* validation in rat models using qPCR, ELISA and gut microbiota sequencing, reporting inflammatory activation and gut microbiota alterations that showed partial concordance with the genetic findings (26). The authors concluded that these findings support a genetically determined microbiota–inflammation axis and identify circulating inflammatory proteins as mediators to prioritize for mechanistic study. While recent bidirectional Mendelian randomization analyses have identified direct causal links between gut microbiota taxa and intervertebral disc degeneration (27, 28), they did not assess whether classic inflammatory immune factors mediate these pathways; in one such study, the phylum Bacteroidetes remained associated with a higher risk of IVDD after Benjamini–Hochberg correction (FDR-corrected P = 0.0365) (28). In a two-sample mediation MR study of 211 gut microbial taxa, 41 inflammatory cytokines and IVDD (FinnGen, 29,508 cases and 227,388 controls), 11 taxa showed suggestive causal associations at nominal significance, of which only the Eubacterium coprostanoligenes group approached the Bonferroni-corrected threshold (P = 3.0×10^−5^); two-step MR revealed no statistically significant indirect effect of cytokines linking gut microbiota to IVDD (20). A null indirect effect does not establish that an alternative, untested pathway is responsible. By contrast, mediation analyses restricted to circulating metabolites have yielded significant indirect effects (13, 17), indicating that null cytokine mediation and positive metabolite mediation can coexist rather than being mutually exclusive.

The specific causal association effect values for each taxon and IVDD are detailed in **Table 1**.

Clinical cohort data on circulating cytokines and low back pain severity are available, but as cross-sectional observations they cannot establish causal direction. In a cross-sectional cohort of 1,007 individuals with chronic low back pain (plasma available from 936), circulating IL-6, IL-1ra and leptin were higher with greater pain and disability severity and TNF was higher with greater disability severity, whereas IFN-γ, IL-15 and IL-10 showed no differences across categories; the authors called for longitudinal and mechanistic studies to clarify whether cytokines serve as diagnostic, prognostic or phenotyping markers (30). Interventions that attenuate disc degeneration can also act without a measurable systemic correlate: in microbiota-depleted mice, Akk treatment reduced local disc inflammation markers (IL-6, TNF-α) and senescence (p16Ink4a), whereas serum IFN-γ, TNF-α, IL-6, IL-2 and IL-10 did not differ significantly from vehicle, and systemic inflammatory cytokines and serum biochemistry were unaltered (18). Conversely, in a lumbar annulus puncture rat model, serum IL-6, TNF-α and IL-1β were all elevated relative to sham animals (31). Whether local and systemic inflammatory compartments are dissociated therefore appears to be model- and direction-dependent, and requires paired, time-resolved sampling. Genetic studies of circulating inflammatory proteins also confirm that the association between inflammatory immune factors and disease shows marked tissue specificity, and local lesions cannot be simply summarized as systemic inflammation (32).

The protective effects of *Akkermansia muciniphila* and *Eubacterium coprostanoligenes* were not statistically explained by the systemic inflammatory immune measures assessed, which is consistent with, but does not prove, independence from systemic inflammatory regulation. In mice administered Akk or Akk-EVs, serum levels of IFN-γ, TNF-α, IL-6, IL-2 and IL-10 showed no significant intergroup differences compared with vehicle controls, yet intervertebral disc degeneration was significantly attenuated across multiple IVDD models, and pharmacologic inhibition of Akk EV secretion abolished these protective effects without evoking systemic cytokine alterations (18). Purified Akk membrane protein did not stimulate macrophages to secrete pro-inflammatory factors *in vitro* (33). This observation is restricted to macrophages *in vitro*; other immune cell types and *in vivo* systemic responses were not assessed, so it does not establish that the protective effect is unrelated to systemic immunity. Relying solely on the circulating inflammatory immune pathway is completely insufficient to explain the nucleus pulposus protective phenotype of these two protective strains, and there is an urgent need to explore microbe-specific local regulatory pathways independent of systemic immunity.

The failure of the circulating inflammatory factors tested to show significant mediation motivates exploration of local, disc-centered immune regulatory mechanisms; it does not establish that such mechanisms operate independently of systemic immunity. Microbiome and metabolome profiling of lumbar cartilaginous endplates in patients with Modic changes documented reduced α-diversity and a distinct microbial composition relative to controls, together with 26 differentially abundant genera, 26 differential metabolites and alteration of the unsaturated fatty acid pathway (34). Circulating cytokines were not measured. As a cross-sectional comparison, these findings are consistent with, but do not prove, local immune activation that is independent of systemic inflammation.

Multivariable Mendelian randomization (MVMR) studies have further identified the mediating role of non-inflammatory blood metabolites: A 2025 MVMR study by Zheng Y-P et al. identified four classes of non-inflammatory blood mediators, with mediation proportions of 7.77%–12.56%, and none of the mediating pathways involved classic circulating inflammatory immune factors such as TNF-α and IL-6 (17). We note that these proportions are numerically similar to those reported for cytokine mediation (7.55%-13.22%), and we therefore apply the same cautious interpretation to both rather than treating one as negligible and the other as conclusive. Existing mediation MR analyses in IVDD have largely centered on inflammatory factors and local microenvironmental signals (35).

The methodological limitations of existing Mendelian randomization studies can also partially explain the negative results for inflammatory immune mediation. These include limited instrument strength for microbial taxa, modest statistical power for indirect effects, incomplete coverage of the circulating mediator repertoire, horizontal pleiotropy, heterogeneity in IVDD phenotype definition across GWAS, and incomplete correction for multiple testing. On the one hand, MR studies only detect peripheral circulating cytokines and completely fail to capture hidden immune regulatory mediators such as local disc metabolites and extracellular vesicles, creating an inherent flaw of omitted mediator variables. Methods such as MR-Egger regression can only correct for instrumental variable bias and cannot address selection bias in mediator indicators (36). On the other hand, the study populations of microbial GWAS databases such as MiBioGen are predominantly European white, lacking genetic data from East Asian and African populations, leading to ethnic stratification bias in the extrapolation of MR conclusions. The negative conclusion of inflammatory immune mediation still requires replication in multi-ethnic cohorts (29). *In vitro*, L-BAIBA suppressed TNF-α-induced extracellular matrix degradation, apoptosis and pyroptosis in nucleus pulposus cells via AMPKα (37). This shows that a locally acting metabolite can modify catabolic and cell-death responses downstream of TNF-α; it does not by itself demonstrate independence from systemic immunity. Classic methodological studies of MR statistics have also made clear that mediation analyses including only circulating indicators cannot cover tissue-specific hidden immune regulatory pathways (38).

## 3 Regulation of local intervertebral disc immune homeostasis by microbial soluble metabolites

### 3.1 The short-chain fatty acid pathway

Short-chain fatty acids (SCFAs) mainly include three core products: butyric acid, propionic acid and acetic acid, synthesized by butyrate-producing microbiota such as intestinal *Clostridium* and *Ruminococcaceae*. Their core mechanisms include histone deacetylase (HDAC) inhibition and GPR41/43 receptor activation, which can directly regulate the local nucleus pulposus immune microenvironment, cell autophagy and extracellular matrix synthesis; anti-inflammatory effects are only a secondary additional effect (39). *In vitro* experiments using human degenerated intervertebral disc tissues confirm that sodium butyrate significantly inhibits the activation of the NF-κB signaling pathway, reduces the secretion of pro-inflammatory factors including TNF-α and IL-1β, and downregulates the expression of matrix-degrading enzyme MMP13, thereby alleviating inflammatory matrix degradation in the degenerated disc, with clear matrix protection and local immune homeostasis regulatory effects (40). This metabolic regulatory effect is consistent with the protective effect of butyrate-producing microbiota identified in Mendelian randomization analysis, and MR studies confirm that butyrate-producing bacteria regulate IVDD risk via blood butyrate levels, an effect independent of circulating inflammatory immune factors (15, 17).

SCFAs provide acetyl-CoA substrates to promote histone acetylation and inhibit HDAC family enzyme activity, regulating the transcription of nucleus pulposus cell senescence and immune-related genes. This epigenetic regulatory pathway is not explained by the TLR4-NF-κB systemic inflammatory immune axis, although direct tests of independence are lacking (39). Homologous studies in bone tissue confirm that SCFAs systemically regulate bone chondrocyte homeostasis and the local immune microenvironment, inhibiting bone resorption and promoting matrix synthesis, through mechanisms that do not rely on the release of inflammatory factors from peripheral immune cells, providing homologous tissue support for the SCFA local immune regulatory mechanism in intervertebral discs (41). Studies in postmenopausal populations show that women who respond to prune intervention to maintain hip bone mineral density have significant differences in gut SCFA-producing microbiota, further supporting the bone metabolism and immune regulatory roles of SCFAs (42). It should be noted that current targeted metabolome studies of Modic endplates have not covered the detection of butyric acid, propionic acid and acetic acid, and relevant conclusions are only derived from background literature references and still require validation in subsequent clinical samples.

### 3.2 The Tryptophan-Trp-AhR local immune regulatory axis

Intestinal *Clostridium* species metabolize dietary tryptophan into indole derivatives such as indolepropionic acid (IPA) (12). These substances can penetrate the intestinal mucosal barrier and vertebral endplate barrier to reach the local intervertebral disc, and are hypothesized to induce regulatory T cell polarization and inhibit the IL-23/IL-17 local pro-inflammatory immune microenvironment. Mechanistic studies in bone tissue demonstrate that IPA derived from *Clostridium sporogenes* activates the pregnane X receptor to suppress osteoclast differentiation and pathological bone resorption. As a tissue-specific microbial immunomodulator, indole metabolites can penetrate the impaired endplate barrier and are hypothesized to regulate the local pro-inflammatory microenvironment of the intervertebral disc, which awaits direct validation in nucleus pulposus experiments (12). Multivariable MR studies show that histidine mediates the protective effect of *Lachnoclostridium* on IVDD, with a mediation proportion of 18.1%, and that this pathway does not involve the systemic inflammatory immune factors tested (13). Of note, histidine is not a downstream metabolite of tryptophan, an important distinction from the indole derivatives discussed above.

The kynurenine pathway, the principal route of tryptophan catabolism, has been implicated in neuropathic pain and other chronic pain conditions (43). Tryptophan-derived indole can stimulate GLP-1 secretion from colonic enteroendocrine L-cells and activate vagal afferent signaling in rats (44). Faecalibacterium prausnitzii and its butyrate production attenuate renal inflammation and dysfunction via GPR43 in a chronic kidney disease model (45). Gut-derived indole-3-acetic acid activates AhR and attenuates neuroinflammation and retinal ganglion cell loss in a glaucoma model (46); whether the same tissue-specific regulatory logic operates in the intervertebral disc has not been tested.

Modic endplate metabolome studies have identified altered local fatty acid metabolism (34); whether tryptophan-derived metabolites are altered in Modic endplates has not been established. In cancer-associated muscle atrophy, serum tryptophan concentration correlates positively with skeletal muscle volume, and a tryptophan-deficient diet reduces myofiber diameter in mice, indicating that tryptophan availability regulates muscle mass (47). The relevance of this axis to disc or endplate degeneration is untested.

### 3.3 The bile acid-GPBAR1 pathway and *E. coprostanoligenes*

*Eubacterium coprostanoligenes* has a specific cholesterol reduction metabolic function, converting dietary cholesterol into coprostanol, while participating in the production of secondary bile acids that are hypothesized to activate the nucleus pulposus GPBAR1/TGR5 receptor to inhibit local matrix degradation and immune activation. This cholesterol-metabolizing strain is a core protective genus identified by MR, and its biological effects depend on bile acid signaling pathway transduction (15, 17). Host primary bile acids must be modified by intestinal anaerobic bacteria to generate secondary bile acids, and *Eubacterium* species are key transforming taxa; their deficiency leads to a significant decline in bile acid signaling pathway activity (48, 49). *Clostridium* probiotics can reshape the bile acid pool and improve metabolism-related organ degeneration and local immune disruption, with potential value for intervention in spinal diseases (50).

MR analysis suggests that cholesterol metabolism-related microbiota are significantly associated with IVDD risk, and the mechanism relies on tissue-specific immune regulation by secondary bile acids. Existing evidence from homologous tissue studies indicates that bile acid receptor activation can inhibit the transcription of MMP family degrading enzymes, slowing disc matrix loss and local immune activation, which remains to be directly verified in intervertebral disc tissue (13, 15). Studies on irritable bowel syndrome show that cholestyramine can improve bone and muscle loss by regulating bile acid metabolism, further corroborating the bone metabolism and immune regulatory roles of the bile acid pathway (48). Neuropathic pain studies have also found that alterations in gut microbiota and metabolite profiles are accompanied by changes in pain phenotypes, with bile acid metabolism serving as an important immunomodulatory link (51). It should be noted that neither current endplate metabolome nor lumbar MR studies have detected bile acid profiles, and relevant conclusions still require validation in subsequent experiments.

### 3.4 Unsaturated fatty acids and the synergistic immune regulatory network of three classes of metabolites

Targeted metabolome studies of Modic endplates provide local clinical evidence for endplate metabolic alterations that are not accompanied by changes in the circulating cytokines measured. Studies show that in the Modic group, 8 unsaturated fatty acids and 4 carnitines are significantly upregulated in the endplate, while 7 metabolites are significantly downregulated. Pathway enrichment shows that differences are mainly concentrated in unsaturated fatty acid metabolism, the malate-aspartate shuttle, and the citric acid cycle. Microbiota correlation analysis shows that Caulobacteraceae is positively correlated with fatty acid levels, while *Blautia* and *Bifidobacterium* are negatively correlated with fatty acid levels, together forming a bacteria-metabolism-immune regulatory axis in the local endplate (34).

Arachidonic acid is metabolized through cyclooxygenase, lipoxygenase and cytochrome P450 pathways to eicosanoids that are generally pro-inflammatory (52). Multiple polyunsaturated fatty-acid-derived lipid mediators can amplify local inflammatory responses within degenerative intervertebral disc tissue (53). Direct experimental evidence specifically validating adrenic acid activity inside disc tissue remains absent. Unsaturated fatty acid metabolism was among the pathways most altered in Modic endplates (34). Direct evidence that these lipid mediators exert local effects within the disc or endplate independent of changes in circulating inflammatory proteins is lacking.

Three classes of microbial metabolites—short-chain fatty acids, tryptophan indole derivatives, and secondary bile acids—are proposed to act via epigenetic immune regulation, local immune modulation and receptor activation, respectively, to influence disc matrix protection (15, 17, 39). Whether these constitute a complete and independent protection network is not established. Circulating inflammatory immune proteins do not fall into the category of microbial small-molecule metabolic mediators and cannot explain the core protective effects of these three classes of metabolites (20). Unsaturated fatty acid disruption in Modic endplates, together with the three metabolic pathways described above, may constitute a local immune-metabolic regulatory system that is not captured by the circulating cytokines measured to date.

## 4 Bacterial extracellular vesicles: cross-barrier immune messengers between the intestinal mucosa and intervertebral disc

Bacterial extracellular vesicles (EVs) are one candidate carrier linking the intestinal mucosa and the spine. This is not obligatory: SCFAs, bile acids and indole derivatives can also circulate freely or bound to albumin, and EV-independent delivery remains plausible. The intervertebral disc is an avascular tissue, and free small-molecule metabolites are easily diluted by blood and cleared by circulating enzymes, making it difficult for them to stably reach the nucleus pulposus. Bacterial EVs can encapsulate metabolites and functional proteins and have been proposed as stable long-distance signal carriers (54, 55). Research on the gut-bone axis also confirms that bacterial EVs can act as core mediators for gut microbiota to regulate local bone immune homeostasis, providing a homologous reference for the disc EV immune pathway (54).

Extracellular vesicles are likely potential immune mediator variables that were not detected in previous cytokine-centered Mendelian randomization studies. All previous Mendelian randomization studies only detected free circulating cytokines, completely ignoring composite immune mediator variables such as proteins and metabolites encapsulated in vesicles. This is one of the core explanations for the negative systemic inflammatory immune mediation results (9, 36). The particle size and molecular composition of extracellular vesicles determine that they cannot be captured by conventional circulating inflammatory immune detection methods, making them plausible candidate mediators that were not captured by cytokine-centered MR studies.

Extracellular vesicles derived from *Akkermansia muciniphila* are currently the most well-studied protective immune vesicles for intervertebral discs. Animal studies show that orally administered Akk-EVs can cross the intestinal mucosal barrier, and targeted enrichment in mouse intervertebral disc tissues has been verified, while EVs derived from *Escherichia coli* show no spinal homing property, supporting the view that Akk-EVs have cross-barrier targeted transport capacity (18, 55). Proteomic screening shows that Akk-EVs carry the B2UKX5 protein (encoded by the *Amuc_1426* gene, molecular weight 31.5 kDa). *In vitro* treatment of TNF-α-injured nucleus pulposus cells with recombinant protein upregulates COL2A and ACAN expression and downregulates MMP13 and CDKN1A expression, regulating local immunity and matrix homeostasis without cytotoxicity (18, 56). Inhibitor experiments further verify the necessity of EVs: after inhibiting vesicle secretion from Akk bacteria, the protective effects of oral Akk bacteria on intervertebral disc degeneration in multiple IVDD models are abolished, indicating that EVs are an essential mediator for the local immune protective effect of Akk bacteria (18).

EVs secreted by different genera carry heterogeneous protein and miRNA contents, which can target and regulate the 3’UTR of nucleus pulposus MMP and ADAMTS genes, inhibit the sustained transcription of matrix degrading enzymes, and reshape the local immune microenvironment. Host nucleus pulposus cells also release exosomes that shape the local immune microenvironment: exosomes from degenerated nucleus pulposus cells carry miR-27a-3p and drive M1 macrophage polarisation, aggravating degeneration *in vivo* (57). Whether host and microbe-derived vesicles interact directly has not been demonstrated. Next-generation sequencing of herniated disc specimens detects a disc microbiota (58), but this does not establish gut origin, EV-mediated transport, or translocation across the endplate.

Bacterial extracellular vesicles can carry small-molecule metabolites, suggesting that vesicle-mediated and metabolite-mediated signaling may intersect rather than operate independently. Direct evidence for such an intersection comes, however, from the gut–bone (54) and gut–joint (55) axes; whether the two act synergistically within the intervertebral disc has not been tested. Evidence from other organ systems supports that fecal microbiota transplantation can reshape the circulating extracellular vesicle pool: in a germ-free mouse FMT model, circulatory extracellular vesicles isolated after transplantation of microbiota from mice with pressure-overload remodeling enlarged primary mouse cardiomyocytes *in vitro* and aggravated hypertrophy and fibrosis *in vivo*, with metagenomic and miRNA sequencing identifying characteristic gut microbes and vesicle-associated miRNAs (59). Direct evidence in bone or intervertebral disc is lacking, and the contribution of donor EVs to FMT efficacy in IVDD remains untested. Metabolically augmented microbial formulations illustrate the feasibility of this approach: oral synbiotic microspheres designed to enhance microbial short-chain fatty acid production improved intestinal barrier integrity and suppressed bone loss in a postmenopausal osteoporosis model (60); analogous formulations have not been evaluated in the intervertebral disc. Reviews of microbiome-based therapeutics classify the beneficial products or effects produced by bacterial strains—termed microbiome mimetics—as one emerging therapeutic class alongside live biotherapeutics (61), but their comparative safety and suitability for spinal applications remain to be established.

Clinical data show that circulating Akk-EV concentrations are inversely correlated with Pfirrmann grade and age, and that Akk-EV and B2UKX5 levels in both circulation and disc tissue are negatively correlated with IVDD severity (18). More broadly, the feasibility of circulating markers for disc degeneration is supported by a serum RNA sequencing study that identified 73 differentially expressed circulating miRNAs in patients with lumbar disc herniation, with a three-miRNA signature (miR-766-3p, miR-6749-3p and miR-4632-5p) proposed as a non-invasive diagnostic biomarker; this was a small, non-randomized preliminary analysis without an age-matched control group (62). Whether circulating vesicle or miRNA signals can serve as markers of degeneration severity, or whether loss of vesicle signals contributes to multi-segment degeneration, requires prospective validation. Currently, neither large-scale MR studies nor endplate metabolome studies have conducted systematic EV tracing or local immune validation experiments, and relevant *in vivo* functional validation remains an evidence gap, representing an important direction for future research.

## 5 Endplate barrier disruption and *in situ* immune remodeling driven by gut bacteria

In addition to soluble metabolites and bacterial extracellular vesicles, bacterial cell-wall components (such as peptidoglycan and lipopolysaccharide), bacterial nucleic acids, and—less certainly—viable bacteria themselves may reach the disc across a damaged endplate. We use “bacterial fragments” hereafter to denote non-viable bacterial material; viability is seldom demonstrated in disc specimens. Vertebral endplate defects and higher Modic scores are more frequent at levels with adjacent disc degeneration than at levels without it (64), consistent with a compromised barrier permitting exchange between the vertebral body and the disc. Whether gut-derived fragments transit this route has not been directly demonstrated. Early studies already confirmed that Propionibacterium acnes in the intervertebral disc can form biofilms, providing histological evidence for bacterial colonization and local immune activation (63). An early randomized controlled trial reported benefit from antibiotic treatment in patients with type 1 Modic changes (MCs) (65). This has not been replicated: the AIM trial found that three months of amoxicillin did not provide a clinically important benefit over placebo (between-group difference in Roland-Morris Disability Questionnaire score at one year, -1.6; 95% CI -3.1 to 0.0; P = 0.04), with drug-related adverse events in 56% versus 34% of participants (66), and a more recent randomized trial of amoxicillin-clavulanate in chronic low back pain with disc herniation was likewise null (adjusted difference in pain at one year, 0.06; 95% CI -0.58 to 0.7) (67). Antibiotic response therefore cannot be presented as indirect proof of bacterial causation.

Type I/II Modic changes are accompanied by collagen rupture and increased microvascular density in the vertebral endplate, and the permeability of this barrier is elevated. We use “mucosal-like” only as a functional analogy: the endplate is a cartilage-bone interface rather than an epithelium, lacks a mucus layer and a mucosa-associated lymphoid compartment, and the analogy should not be extended beyond barrier permeability, providing an anatomical basis for hematogenous translocation of bacterial fragments. Bacterial components such as LPS and peptidoglycan may activate the TLR2/4 pathway locally within the intervertebral disc, a scenario hypothetically compatible with a lack of synchronous elevation of peripheral serum TNF-α or IL-6. This hypothetical tissue-specific local immune remodeling provides one plausible interpretive framework for the negative mediation findings from Mendelian randomization studies (34).

Estrogen has a protective effect on the endplate barrier: 17β-estradiol maintains endplate barrier immune isolation function and reduces the translocation efficiency of bacterial fragments. Declining estrogen levels after menopause has been proposed as a trigger for endplate barrier damage. It has been hypothesized that menopause adversely affects vertebral endplate quality and reduces nutrient diffusion to the disc, a hypothesis the authors stated requires confirmation by post-contrast MRI studies (69). In ovariectomized rat models, 17β-estradiol supplementation alleviated intervertebral disc degeneration by inhibiting NF-κB signaling (68). Direct measurements of endplate thickness or porosity in postmenopausal women were not identified in the literature we examined. During aging, endplate chondrocyte apoptosis increases, matrix mineralization is dysregulated, and the diffusion capacity of nutrients and macromolecules decreases year by year, leading to a linear increase in the risk of bacterial fragment translocation.

The human body has both a peripheral circulating immune system and local immune compartments. After degeneration, the immune privilege of the intervertebral disc is lost, and local TLR pathways can be activated autonomously (70). Macrophages, dendritic cells and T cells resident in the nucleus pulposus form a local immune network. Under physiological conditions, these cells maintain immune privilege with an anti-inflammatory M2 phenotype and regulatory T cell dominance. Once the physical barrier between the nucleus pulposus and the systemic circulation is breached, immune cell infiltration occurs and the nucleus pulposus is exposed as a foreign antigen; local macrophages polarize toward the pro-inflammatory M1 phenotype and the Th17/Treg balance is disrupted, driving sustained low-grade inflammation and matrix degradation (71). Whether this local response is quantitatively independent of peripheral immune cell recruitment has not been established.

Whether bacteria detected in disc specimens are true commensals, contaminants, or translocated from the gut remains unresolved. Next-generation sequencing of normal and degenerated discs found that the Modic group was dominated by gram-negative taxa (more than 50% of OTUs), with opportunistic gram-negative organisms enriched relative to non-Modic discs, and that bacterial metabolites and quorum-sensing molecules supported colonization rather than contamination (72). *In vitro*, Cutibacterium acnes stimulate nucleus pulposus cells to upregulate IL-1β, IL-6, IL-8 and inducible nitric oxide synthase, an effect partially reduced by TLR2/4 inhibition in three of five donors (73). By contrast, a multicenter surgical study comparing adults undergoing discectomy with adolescent scoliosis controls found that C. acnes detection was similar in both groups (72% versus 70%), that 235 of 240 disc and vertebral samples were negative for bacterial DNA, and that bacterial findings were not associated with Modic changes—findings that favor contamination and have been used to question antibiotic treatment (74). Ribotyping of isolates from herniated discs identified phylogroups, notably types II and III, that are infrequently recovered from skin, indicating that the organism’s role should not be readily dismissed, although this does not itself establish a gut origin (75). Low-virulence anaerobic bacterial fragments are mostly detected in young adult disc herniation samples, with no systemic sepsis or elevated inflammatory immunity, only inducing local chronic low-grade degenerative stimulation, further supporting the local translocation immunity hypothesis.

In aging mice, intestinal tight junction protein expression is downregulated and intestinal mucosal permeability increases, leading to elevated circulating bacterial peptidoglycan concentrations, which simultaneously aggravate vertebral endplate injury and local inflammation, forming a vicious cycle of “intestinal barrier damage → endplate barrier injury → local immune activation”. These observations are derived from animal models and remain to be validated in human subjects.

We propose that three classes of signal—soluble microbial metabolites, Akk-derived extracellular vesicles, and bacterial material reaching the disc across a damaged endplate—may jointly influence the synthesis/degradation balance of the nucleus pulposus matrix and local immune homeostasis. Whether they act synergistically, and whether they are quantitatively important in human IVDD, is not established; this proposal therefore offers one possible, not definitive, reconciliation of the null mediation findings from Mendelian randomization studies. The core characteristics and evidence levels of the three-candidate circulating-cytokine-independent immune regulatory pathways of the gut-disc axis are summarized in **Table 2**.

**Table 2:** Core characteristics of the three candidate circulating-cytokine-independent immune regulatory pathways of the gut-disc axis.

Pathway type	Key effector molecules	Target of action	Level of evidence	Key supporting references
Soluble metabolite pathway	Short-chain fatty acids	HDAC/GPR41/43, nucleus pulposus matrix synthesis and inflammatory inhibition	*In vitro* disc experiments + genetic suggestive evidence	(15, 17, 39, 40)
	Tryptophan indole derivatives	AhR, local immune regulation	Homologous tissue reference + genetic suggestive evidence	(12, 43, 46)
	Secondary bile acids	GPBAR1, matrix degradation inhibition	Homologous tissue reference + genetic suggestive evidence	(48–50)
	Unsaturated fatty acids	Local lipid metabolism, endplate inflammation regulation	Direct clinical metabolome evidence	(34, 52)
Bacterial extracellular vesicle pathway	Akk-EV carrying *Amuc_1426* protein	MMP/COL2A, nucleus pulposus matrix homeostasis	Animal experiments + *in vitro* disc experiments	(18, 55, 56)
	EV-encapsulated small-molecule metabolites	Synergistic metabolic regulation, nucleus pulposus senescence	Homologous evidence + mechanistic deduction	(55, 61)
Bacterial local translocation pathway	Low-virulence anaerobic bacterial fragments	TLR2/4, local immune activation	Direct clinical sample evidence	(72, 73, 75)
	Endplate barrier damage	Tight junction proteins, anatomical basis for bacterial translocation	Animal experiments + clinical imaging evidence	(34, 69)

Hierarchy of evidence from highest to lowest: direct clinical evidence > *in vivo*/*in vitro* disc experimental evidence > homologous tissue reference evidence > genetic epidemiological suggestive evidence.

## 6 Methodological limitations and translational prospects of mucosa-targeted immune interventions

### 6.1 Multidimensional methodological limitations

Current research on the gut-disc axis has multidimensional methodological shortcomings. A particular concern is that evidence is drawn from distinct compartments—fecal, mucosa-associated, circulating, endplate and disc—that are frequently treated as continuous without demonstration of biological continuity. Detection of bacterial material in a low-biomass disc sample does not establish gut origin; claims of translocation require paired, strain-resolved sampling with stringent contamination controls. The various limitations and optimization directions are summarized in **Table 3**. First are the limitations of population genetic studies: Mendelian randomization studies only detect peripheral circulating inflammatory immune factors and completely lack multi-omics data on local disc transcriptomes, immune cell subsets and metabolomes. GWAS databases such as MiBioGen and FinnGen are predominantly European populations, with significant ethnic stratification bias. There is also a lack of functional validation experiments in humanized mice colonized with protective genera such as *Akkermansia* and *E. coprostanoligenes* (16, 17, 36). Although the FinnGen study provides genetic data from an isolated population with well-characterized phenotypes, it is still predominantly European, and its applicability to East Asian populations remains to be verified.

**Table 3:** Key methodological limitations of current research and corresponding optimization directions.

Limitation category	Specific issues	Optimization strategies	Supporting references
Population genetic research	Only European population data; mediator indicators limited to circulating inflammatory factors; lack of local multi-omics data	Conduct multi-ethnic cohort studies; include metabolites/EVs as mediator variables; supplement spatial multi-omics and immune cell profiling of the intervertebral disc	(16, 29, 36)
Microbial sequencing	Low-biomass samples are susceptible to reagent contamination; no unified correction workflow	Implement strict blank negative controls; establish standardized contamination correction workflows	(58, 72, 74)
Study design	Mostly cross-sectional studies; unable to clarify causal temporal sequence	Conduct long-term longitudinal follow-up cohorts; design interventional validation studies	(9)
Mechanistic research	Mostly homologous tissue references; lack of direct functional validation in the intervertebral disc	Construct disc-specific cell models; conduct *in vivo* targeted intervention experiments	(10, 41, 46)

Second are limitations in study design and sequencing methods: most existing clinical evidence adopts a cross-sectional case-control design, which can only collect microbiota and degeneration indicators simultaneously and cannot distinguish whether microbial dysbiosis is the cause or a secondary change of degeneration, lacking longitudinal temporal cohort evidence (9). The intervertebral disc is a low-biomass sample, and 16S rRNA sequencing is highly susceptible to contamination from kit background microbiota and environmental reagents; next-generation sequencing approaches such as those applied to herniated disc specimens (58) require the same rigorous controls. Most current clinical cohorts do not set strict blank negative controls, leaving the reliability of results in question. Interspecific crosstalk of quorum sensing signals may also interfere with the qualitative results of low-biomass microbiota. Although next-generation sequencing technology has been applied to disc microbial detection, a standardized workflow for contamination correction has not been unified.

### 6.2 Potential directions for mucosa-targeted microbial immune interventions

Four directions have been proposed for the gut-disc axis: fecal microbiota transplantation (FMT), specific probiotic supplementation, SCFA/bile acid metabolic precursor drugs, and engineered Akk-EV postbiotic preparations. In a rat model of intervertebral disc degeneration, fecal microbiota transplantation reversed the elevations of TNF-α, IL-1β, IL-6, MMP-3, MMP-13, NLRP3 and Caspase-1 and the reductions in collagen II and aggrecan, and increased gut microbiota diversity and abundance (76). Reviews of the gut-disc axis likewise describe dysbiosis-associated increases in intestinal permeability, with translocation of bacterial metabolites such as short-chain fatty acids and lipopolysaccharides, and report that microbiota-restoring interventions including fecal transplantation reduce inflammation in animal models (56). All remain experimental in IVDD. Drawing on protective microbiota screened by MR, stratified and precise interventions for people with low back pain can be developed around mucosal barriers and local immune regulation (61, 77).

Prebiotics can selectively enrich protective microbiota such as butyrate-producing bacteria, *Eubacterium* and *Akkermansia*, upregulate beneficial metabolite concentrations, repair the intestinal mucosal barrier, and have potential for long-term oral intervention in chronic low back pain. However, most existing randomized controlled clinical trials of probiotics focus on people with comorbid anxiety and depression, and large-scale trials targeting simple intervertebral disc degeneration and Modic changes are very scarce. Animal experiments show that specific probiotics can regulate gut microbiota in mice, reduce nucleus pulposus MMP expression, and alleviate puncture-induced disc herniation injury, providing animal-level support for probiotic mucosal immune intervention.

Engineered microbial vesicles loaded with metabolites to improve targeting represent an important direction for postbiotic research, and can improve local immune regulation efficiency via mucosal delivery (60). m6A methylation can regulate nucleus pulposus cell senescence—METTL3 was upregulated in annulus puncture-induced rat disc degeneration and modulated TLR2 m6A modification (31). Epigenetic regulators such as m6A may represent potential combinatorial therapeutic targets alongside microbiota-directed strategies, although direct links between gut microbiota and disc m6A modification remain uncharacterized.

However, clinical translation still faces multiple challenges: live probiotics have limited colonization efficiency in the gut and cannot stably increase the abundance of target strains; unmodified bacterial EVs lack disc targeting and are easily cleared by the reticuloendothelial system; and long-term microbial intervention may disrupt the stability of the gut ecosystem itself. Stratified intervention based on patient endplate barrier status and gut microbiota composition has been proposed, but its safety, manufacturing, biodistribution, ecological effects and clinical efficacy are unresolved and it should not be presented as imminent.

## 7 Discussion

Although the three-pathway conceptual model presented in this review can reconcile observed dissociation between peripheral inflammatory markers and clinical low-back-pain phenotypes (30, 34), several critical unresolved controversies and knowledge gaps remain in gut-disc-axis research and deserve emphasis.

Supporting clinical imaging observations have shown that vertebral-endplate defects and higher Modic scores occur more frequently at spinal levels adjacent to degenerated discs (64).

Foremost among these is the origin of microbial signals detected within degenerated disc specimens. Despite multiple reports identifying bacterial signatures in degenerative disc tissue, low-biomass sequencing cannot fully exclude surgical-skin or kit-reagent-derived contamination (58, 72). While ribotyping and biofilm-functional phenotyping of Cuti-bacterium acnes offer a potential approach to distinguish gut-derived from skin-derived isolates, definitive validation of the bacterial translocation hypothesis still requires studies with strict contamination-control workflows and prospective longitudinal cohorts (74, 75).

Sex dimorphism represents another largely under-investigated dimension in this field. It has been hypothesized that post-menopausal estrogen declines compromise vertebral endplate barrier integrity and elevates susceptibility to severe IVDD and Modic-change-related pathology (68, 69). However, direct human evidence supporting this causal chain remains absent. Future mechanistic and clinical studies should explicitly incorporate sex as an important biological variable; sex-stratified analyses will also be valuable when evaluating mucosa-targeted microbial immunotherapies for chronic low-back pain.

## 8 Conclusion and perspectives

Mendelian randomization studies to date have not identified significant mediation by the systemic circulating inflammatory immune factors tested, which challenges, but does not exclude, the traditional single inflammatory hypothesis as the sole explanation for the gut microbiota-IVDD association. Three candidate pathways-soluble microbial metabolites, microbial extracellular vesicles, and bacterial material reaching the disc across a damaged endplate-are proposed to influence nucleus pulposus matrix homeostasis and the local immune microenvironment. Whether they act synergistically, and whether they are quantitatively important in human IVDD, is not established by the available evidence. The more defensible contribution of this review is a critical update that incorporates recent MR findings and the 2026 *Akkermansia* EV study, rather than an entirely new framework. The two core protective genera *Eubacterium coprostanoligenes* and *Akkermansia muciniphila*, identified from MR population studies, are supported by both population epidemiological evidence and cellular and molecular immune mechanisms, providing a rationale for investigating microbial-targeted immunotherapy of chronic low back pain, while recognizing that the supporting evidence remains largely preclinical.

Current research still has shortcomings including single ethnic populations, lack of local immune multi-omics evidence, insufficient *in vivo* EV tracing, and scarce longitudinal cohorts. Future research needs to conduct multicenter, multi-ethnic clinical cohorts, combined with humanized mouse models and spatial multi-omics technology, to further verify the pathological roles of the three local immune pathways and promote the clinical translation of mucosa-targeted microbial immune interventions.
